# Maintenance of Medical Care of Children and Adolescents With Asthma During the SARS-CoV-2/COVID-19 Pandemic: An Opinion

**DOI:** 10.3389/ijph.2022.1604849

**Published:** 2022-05-31

**Authors:** Malik Aydin

**Affiliations:** ^1^ Laboratory of Experimental Pediatric Pneumology and Allergology, Department of Human Medicine, Center for Biomedical Education and Research, School of Life Sciences (ZBAF), Faculty of Health, Witten/Herdecke University, Witten, Germany; ^2^ Center for Child and Adolescent Medicine, Center for Clinical and Translational Research (CCTR), Helios University Hospital Wuppertal, Witten/Herdecke University, Wuppertal, Germany

**Keywords:** public health, pandemic, healthcare, asthma, patient education

The clinical picture of asthma is gaining increasing global attention [1]. Considerable effort is being put into understanding the underlying mechanisms and causes of asthma [2, 3]. Prevention concepts and disease education are some areas receiving increased attention from patients and caregivers in order to catch complications earlier [4, 5]. Indeed, the main goal should be that the patient becomes experienced about his/her disease [5]. However, it is not only in medically underserved regions where children and adolescents can succumb to asthma complications. Here, for example, the case of a 9-year-old girl, Ella Kissi-Debrah, from the United Kingdom, where the court recently ruled that environmental pollution levels in the region of Ella's home were far above the norm and led to her death should not fade into oblivion [6]. She suffered from several asthma attacks before her death. Asthma schooling/patient education are important concepts that can lead to better disease awareness and understanding. In Germany, such asthma training concepts (“Asthmaschulung”) exist for appropriate age groups, in which children and parents are taught in small groups using practical and playful exercises [7]. Themes including understanding the disease, exercises for the early recognition of urgent situations, and the correct use of medications and inhalation techniques, as well as other thematically relevant contents, are the main topics [7]. Such education measures increase the self-confidence of affected patients in dealing with the disease, meaning doctor’s visits may also become less necessary [8]. Unfortunately, the current SARS-CoV-2-/COVID-19-pandemic has not only led to corresponding lockdowns in different areas of society, but has also affected the implementation of asthma education. Virtual training can be complementary to other treatments, and some associations offer training, such as self-education programs, through close-meshed testing [9]. Parents who were involved in these education programs showed better asthma control with their affected children. Importantly, the United Nations has set auspicious goals for the 2030 Agenda for Sustainable Development [10]. Here, important notes on asthma prevention and control are given, which should be taken into serious consideration in asthma care.
